# Positive selection on ADAM10 builds species recognition in the synchronous spawning coral *Acropora*

**DOI:** 10.3389/fcell.2023.1171495

**Published:** 2023-04-20

**Authors:** Masaya Morita, Seiya Kitanobo, Shun Ohki, Kogiku Shiba, Kazuo Inaba

**Affiliations:** ^1^ Sesoko Station, Tropical Biosphere Research Center, University of the Ryukyus, Nishihara, Japan; ^2^ Shimoda Marine Research Center, University of Tsukuba, Shimoda, Japan; ^3^ Department of Immunology, Graduate School of Biomedical and Health Sciences, Hiroshima University, Hiroshima, Japan

**Keywords:** *Acropora coral*, ADAM10, positive selection, fertilization, species recognition, synchronous spawning

## Abstract

The reef-building coral *Acropora* is a broadcast spawning hermaphrodite including more than 110 species in the Indo-Pacific. In addition, many sympatric species show synchronous spawning. The released gametes need to mate with conspecifics in the mixture of the gametes of many species for their species boundaries. However, the mechanism underlying the species recognition of conspecifics at fertilization remains unknown. We hypothesized that rapid molecular evolution (positive selection) in genes encoding gamete-composing proteins generates polymorphic regions that recognize conspecifics in the mixture of gametes from many species. We identified gamete proteins of *Acropora digitifera* using mass spectrometry and screened the genes that support branch site models that set the “foreground” branches showing strict fertilization specificity. ADAM10, ADAM17, Integrin α9, and Tetraspanin4 supported branch-site model and had positively selected site(s) that produced polymorphic regions. Therefore, we prepared antibodies against the proteins of *A. digitifera* that contained positively selected site(s) to analyze their functions in fertilization. The ADAM10 antibody reacted only with egg proteins of *A. digitifera*, and immunohistochemistry showed ADAM10 localized around the egg surface. Moreover, the ADAM10 antibody inhibited only *A. digitifera* fertilization but not the relative synchronous spawning species *A. papillare*. This study indicates that ADAM10 has evolved to gain fertilization specificity during speciation and contributes to species boundaries in this multi-species, synchronous-spawning, and species-rich genus.

## 1 Introduction

In sessile animals such as coral, gamete recognition is a trait associated with mate choice. Species recognition by gametes is crucial for synchronous spawning species, especially species-rich genera (Knowlton, 2000; Carlisle and Swanson, 2021). Gamete recognition (species- and self-recognition) provides a mechanism for mate choice to distinguish self from non-self and species identity in hermaphroditic species that broadcast gamete during multispecies spawning events. Mate choice is imperative for maintaining species boundaries and the fitness of descendants (Gowaty et al., 2007; Harrison and Larson, 2014; Ihle et al., 2015). The mechanism(s) of species- and self-recognition are essential for species boundaries (Willis et al., 2006). However, how gamete fertilizes with conspecifics of the other colonies is still unknown in the *Acropora* corals.

The coral *Acropora* is a broadcast-spawning hermaphrodite. Their gametes are fertilized in the water column after the release of a small package filled with sperm and eggs called “gamete bundles.” In most species, gametes possess strict species recognition to mate with conspecifics after synchronous spawning among congeneric species (“multi-specific spawning”) (Willis et al., 2006; Baird et al., 2009). Although species recognition is a prerequisite for reproductive isolation and species boundary in *Acropora* spp., the mechanism of species recognition is still unclear.

In *Acropora*, synchronous spawning behaviors provoke a risk of hybridization, and therefore, specific fertilization pathways are required for their species boundary. For example, sperm may swim toward eggs (Morita et al., 2006), but the cascades from gamete interaction with conspecifics to membrane fusion are still unknown. In contrast, gamete recognition proteins leading to adhering conspecific gametes are identified. For example, gamete recognition protein “binding” in sea urchins contributes to species-specific adhering. The bindin is under positive selection (Zigler et al., 2005), and genotypes of “bindin” evolved to obtain fertilization efficiency depending on the sperm concentration (Levitan et al., 2007). In addition, the genotypes of the “bindin” changed rapidly according to the fertilization condition due to changes in sea urchin population number (Levitan and Stapper, 2009). Lysin in the sperm of the broadcast-spawning marine invertebrate, abalone, is also involved in the gamete species recognition (Vacquier and Lee, 1993). Lysin is also under positive selection (Lee et al., 1995; Galindo et al., 2003), and interacts with the egg protein VERL (Galindo et al., 2002; Aagaard et al., 2010). The VERL shows coevolution with lysin (Clark et al., 2009). However, gamete proteins involved in *Acropora* fertilization have not been fully studied.

The integrin family of proteins is involved in cell–cell adhesion (Hynes, 1987; Arnaout et al., 2007). In *Acropora*, its involvement in sperm and egg interactions (Iguchi et al., 2007) and its divergence in terms of sequences and expression (Knack et al., 2008) have been reported. The ADAM family of disintegrins and metalloproteases includes ADAM2 (a “fertilin”), which is implicated in sperm–egg binding via integrin—ADAM binding (Evans, 2001; Merc et al., 2021). However, the function of integrins and ADAM in the fertilization of coral have not yet been investigated. Although integrin β1 is partly involved in fertilization in *Acropora*, the involvement of integrins in fertilization in mammalian species with an internal fertilization system is controversial (Miller et al., 2000; He et al., 2003; Barraud-Lange et al., 2007). For example, antibodies against integrins suppress fertilization (Barraud-Lange et al., 2007). Fertilization occurs in eggs (without the ZP) lacking integrin α6β1 (Miller et al., 2000), and the removal of the ZP layer indicates that integrin β1-knockout mice are not sterile (He et al., 2003).

Another family of candidate proteins with a role in sperm—egg interaction is that of the transmembrane tetraspanins. In mammals, the tetraspanin family members participate in primary sperm binding, gamete fusion, and polyspermy blocking (Jankovicova et al., 2020). Of the tetraspanins, the cluster of differentiation (CD9) is essential in the mouse gamete fusion and fertilization (Kaji et al., 2000; Miyado et al., 2000). CD9 participates in the formation of integrin α6β1/tetraspanin clusters in the plasma membrane, which are required for gamete fusion (Ziyyat et al., 2006). The other tetraspanins play many roles, such as sperm-to-egg binding in the ZP (CD9, CD81, and CD151) (Yanagimachi, 1994; Jin et al., 2011) and polyspermy blocking at the plasma membrane (CD9 and CD81) (Talbot and Dandekar, 2003; Ravaux et al., 2018). However, the presence and role of tetraspanins in *Acropora* have not been studied.

Although these proteins in mammals are involved in gamete binding at fertilization, the functions of the proteins in the coral *Acropora* are unknown. Released gametes must mate with conspecifics within the mixture of heterospecifics. In other words, gamete proteins for species recognition must be polymorphic to recognize conspecifics.

The extracellular region of proteins potentially underlies species recognition due to its interaction with other proteins localizing on the gamete’s surface (Swanson and Vacquier, 2002; Palumbi, 2009; Carlisle and Swanson, 2021). The rapid evolution of the recognition sites is supposed to arise via a positive selection of specific codons in the extracellular region. The species-recognition sites are typically diverse, and many proteins are potentially involved in recognition. Taken together, the history of the species-recognition proteins reflects the speciation history of the coral *Acropora*.

In this study, gamete species-recognition proteins in the coral *Acropora* were explored. Proteins in sperm and eggs from *A. digitifera* were identified using mass spectrometry. The rates of molecular evolution of integrins, ADAMs, and tetraspanins from the identified proteins were analyzed to focus on the acquisition of species recognition. In the analyses, we set non-crossing species as those that acquired strict species recognition. Presumably, proteins in the non-crossing species have positively selected sites at the recognition region. The analyses indicated four proteins, ADAM10, ADAM17, tetraspanin 4, and integrin α9 that were candidates for the recognition proteins. The function(s) of the candidates in fertilization were investigated via antibody treatment. Although the positively selected sites in ADAM10 are supposed to be strong enough to be rigor species recognition in the more than 110 species in the coral *Acropora*, the inhibitory effect of the antibody was species-specific. Therefore, ADAM10 could be one of the gamete recognition proteins in the broadcast spawning coral *Aropora*.

## 2 Materials and methods

### 2.1 Coral

*Acropora digitifera*, *A. austera*, *A. tenuis*, and *A. papillare* were used for fertilization analyses. *A. tenuis* and *A. austera* spawn the same night, but the spawning time was earlier than the *A. digitifera*. *A. papillare* spawns closer to the *A. digitifera* and their gametes are compatible (Table 2). The other species, *A. intermedia*, *A. florida*, and *A. donei*, were used for RNA isolation and analysis of cDNA sequences of candidate genes. Published sequence data of 15 *Acropora* species were used, and species were re-sequenced when the registered sequences lacked parts of the open reading frames (ORFs). All colonies were collected at Sesoko Island, Okinawa Prefecture, Japan.

### 2.2 Mass spectrometry (MS) analyses to identify integrins in Acropora

Eggs and sperm of *A. digitifera* were collected after spawning according to the previously described methods (Morita et al., 2006), and proteins in the eggs or sperm were analyzed with liquid chromatography-tandem MS at the Kazusa DNA Research Center (Ibaraki, Japan). To identify proteins, the genome information of *A. digitifera* was used. Approximately 2 g of eggs or 100 mg of sperm were used for the analyses.

### 2.3 Isolation of orthologs of the integrin, tetraspanin, and ADAM families

Orthoscope v1.5.1 for *Acropora* (http://yurai.aori.u-tokyo.ac.jp/orthoscope/Acropora.html) (Inoue and Satoh, 2019) was used to isolate orthologs. To isolate CD9 orthologs in *Acropora*, we used a fasta file of CD9 from *Homo sapiens* (NM_001769.4).

Phylogenetic trees of isolated ORF sequences were constructed using RaxML with a rapid bootstrap and general time reversible-gamma model (Stamatakis, 2006), and the sequences were aligned using MAFFT v. 7 (multiple alignment program for amino acid or nucleotide sequences) (https://mafft.cbrc.jp/alignment/server/) (Katoh et al., 2019). The aligned *phylip* files and the maximum likelihood (ML) tree files were used for molecular evolutionary analyses with CodeML (Yang, 1997).

### 2.4 Molecular evolutionary analysis of candidate genes

The relative rates of synonymous and non-synonymous substitutions in Integrins, Tetraspanins, and ADAMs were calculated using CodeML in PAML (Yang, 1997). Complete ORFs of functional genes from the isolated sequences in Orthoscope were used in the analyses. The codon site model (Model 8 vs. 7) was used, and then confirmed comparison between Model8 and 8a (Supplementary Figure S1), and Bayes empirical Bayes (BEB) analyses were used to detect positively selected sites in the candidate genes (Swanson et al., 2003; Yang et al., 2005).

Branch site analyses (model 2a) were conducted in candidate proteins setting non-crossing species as foreground branches (Yang et al., 2005; Zhang et al., 2005) (Supplementary Figure S1). If the ML model included a category of sites with non-synonymous/synonymous mutations (dN/dS > 1), positive selection likely acted on those sites along that specific lineage. Based on previous studies (Hatta et al., 1999; Fukami et al., 2003; Suzuki et al., 2016), we set non-crossing species (*A. digitifera*, *A. nasuta*, *A. accuminata*, *A. muricata*, *A. hyacinthus*, and *A. cytherea*) as the foreground and crossing species (*A. tenuis*, *A. yongei*, *A. intermedia,* and *A. florida*) as the background. Several species where species-specificity has not yet been identified (such as *A. selago*, *A. microphathalma*, *A. awi*) were set as background branches. In the null model, *dN/dS* of positively selected sites in the foreground was constrained to one. A likelihood ratio test was conducted with one degree of freedom. If the branch site model was supported, positively selected sites calculated from BEB analyses were checked.

We also did branch site analyses with aBSREL (http://www.datamonkey.org/absrel) using phylip file (Smith et al., 2015). We set non-crossing species as foreground branches at sites and run the analyses.

### 2.5 Synthesis of cDNA for construction of expression vectors

Fragments of coral for RNA extraction were collected by snorkeling 3–5 months prior to the predicted spawning month in June. Total RNA was extracted from fresh or preserved coral fragments using TRIzol reagent (Thermo Fisher, Waltham, MA, United States); cDNA was synthesized from the total RNA using SuperScript IV (Thermo Fisher, Waltham, MA, United States) with oligo dT primers.

### 2.6 TA cloning candidate gene cDNA

TA cloning was performed to isolate several genes from *A. papillare* and *A. donei*, whose sequences were not indetified. First, target sequences were amplified with ExTaq (Takara, Ohtsu, Japan) using several primers (Supplementary Table S1). The PCR products were ligated into the pGEM-T Easy Vector (Promega, Madison, WI, United States), which was then transformed into JM109 competent cells (Takara, Ohtsu, Japan). Plasmids were extracted, and cycle sequencing reactions were conducted using ABI BigDye Terminator version 3.1 and Cycle Sequencing Kits with T7 or SP6 primers, followed by capillary electrophoresis in an ABI 3730xl sequencer (Applied Biosystems, Foster City, CA, United States).

### 2.7 Antibody generation

We generated antibodies against the genes that were positively selected in *A. digitifera*. Proteins were expressed with the expression vector, pColdPros2, purified, and then the expressed proteins were used as antigens. To construct expression vectors, primers were designed to cover positively selected sites. The target region was amplified using Primstar HS (Takara, Otsu, Japan) and ligated into the vector after restriction enzyme treatment (XhoI/EcoRI) using the DNA Ligation Kit - Mighty Mix (Takara, Otsu, Japan). The ligated plasmid was subcloned into DH5α cells, and the plasmid was isolated. The isolated plasmid was again transformed into BL21 cells, which were then cultured at 37°C in Luria Bertani medium containing ampicillin until reaching OD_600_ of 0.5, and the expression was induced at 15°C in the presence of 1 mM isopropyl β-D-1-thiogalactopyranoside (IPTG) for 24 h. Expressed proteins were solubilized with 8 M urea and 2 M thiourea and dialyzed against 5 M urea overnight. The supernatant of the extract was applied to TALON resin (Takara, Otsu, Japan). The resin was equilibrated with equilibration buffer (300 mM NaCl and 50 mM NaH_2_PO_4_, pH 7.4) and the proteins eluted with elution buffer (300 mM NaCl, 50 mM NaH_2_PO_4_, and 150 imidazole, pH 7.4). The purified proteins (3–5 mg) were dialyzed against phosphate-buffered saline, generating a polyclonal antibody. Antibody preparation was conducted at Biologica Co. (Nagoya, Japan). Antiserum was purified with Protein A, and IgG was eluted with 0.1 M glycine (pH 3.0). The eluted IgG with glycine was dialyzed with PBS. The IgG concentration was adjusted to 1.0 mg/mL with PBS.

### 2.8 Western blotting

Egg or sperm proteins were subjected to 7.5% or 10% acrylamide gel electrophoresis, and the separated proteins were transferred to polyvinylidene difluoride membranes. The membrane was then blocked with 5% (w/v) skim milk and Tris-base saline (TBS)-Tween (150 mM NaCl, 0.05% (v/v) Tween-20, 25 mM Tris-HCl, pH 7.4) overnight at 4°C. The first antibody reaction was carried out at 1/5,000–1/10,000 dilutions in the blocking solution for 1 h at room temperature (25–27°C), and the membrane was washed with TBS-Tween three times for 10 min each. Then, secondary antibody reactions of horseradish peroxidase (HRP)-labeled anti-rabbit immunoglobulin (Ig)G (Rockland, Limerick, PA, United States) were carried out at 1/20,000 dilution in the blocking solution for 1 h at room temperature. The membranes were again washed with TBS-Tween three times for 10 min each. Protein signals were detected using EzWestLumi Plus (ATTO, Tokyo, Japan).

### 2.9 Immunohistochemistry

Localizations of ADAM10 and Integrin α9 were examined 1 week before spawning according to the method of Morita et al. (2019). Fragments of the coral, *A. digitifera,* were fixed in Bouin’s solution. After the skeletons were dissolved, tissues were embedded in paraffin and cut into 5-μm-thick sections. The sections were washed with 0.1 M phosphate-buffered saline (PBS), soaked in methanol containing 3% H_2_O_2_ for 15 min, and washed with PBS. The H_2_O_2_-treated slides were blocked with goat serum from a Histofine kit (Nichirei, Tokyo, Japan) overnight at 4°C. After blocking, the slides were incubated with an antibody against ADAM10 (1/2000 dilution) in PBS containing 1% (w/v) BSA at 4°C for 4 h, and then washed with PBS for 5 min, three times each. The slides were incubated with biotinylated secondary antibody from the Histofine kit for 15 min at room temperature and then washed with PBS. Streptavidin-horseradish peroxidase (HRP) was applied to the slides for 15 min at room temperature and washed with PBS. Peroxidase activity was visualized with 3,3′-diaminobenzidine (DAB) in a Tris-HCl (pH 7.6) buffer containing H_2_O_2_.

### 2.10 Fertilization experiments

Fertilization trials were carried out with eggs and sperm after spawning of *A. digitifera*, *A. austera*, and *A. papillare*. Eggs were incubated with filtered seawater (SW) with one of the developed antibodies (10 µL antibody (1 mg/mL)/1 mL SW) for 10 min at room temperature, and sperm concentration was adjusted to 10^6^ sperm/mL with filtered seawater. Fertilization was confirmed by observing for developed embryos after 2.5–3 h. Fertilization rates were calculated from ratios of developed embryos and the total number of eggs.

### 2.11 Statistical analysis

Pairwise comparisons using Wilcoxon rank sum test was used to determine significant differences among treatments in fertilization experiments (*p* < 0.05). Bonferroni correction was carried out for multiple comparisons. R v. 4.0.1 was used for the analysis (Team, 2020).

## 3 Results

### 3.1 Integrin, ADAM, and tetraspanin expression in *Acropora* eggs and sperm

Mass Spectrometry (MS) analyses showed that several integrin and ADAM proteins were present in the eggs and sperm (Table 1). Integrin β1 was detected in eggs, and variables of integrin α and β-like (integrin α-X, -V, -9, -PS1, and β-PS) were also found. However, integrins were not found in the sperm. Several ADAM proteins were found in both sperm (ADAM7 and 10) and eggs (ADAM 6, 10, 12, 17, and 18). On the other hand, ADAM10 was detected only in the eggs by western analyses (see below). In addition, bindin, lysin, and VERL contributing gamete species recognitions in the sea urchin and abalone were not found.

**TABLE 1:** Identified ADAM-Integrin and tetraspanin family in sperm and egg proteins.

					Codon site model						Branch site model							aBSREL
Integrins	Gene bank ID	Name of identified proteins	Sperm	Eggs	model8	model8a	model7	ΔlnL	P	Number of BEB selected site	model2b	Null	ΔlnL		P	BEB selected sites		
	XP_015755972.1	PREDICTED: integrin beta-1-like [*Acropora digitifera*]		○	−3775.2	−3776.3	−3776.68	2.1	0.15	2 codon sites	−3776.3	−3776.3	0		1			
	XP_015777528.1	PREDICTED: integrin alpha-V-like [*Acropora digitifera*]		○	−3488	−3503.8	−3503.99	31.4	<0.0001*	4 codon sites	−3503.8	−3503.8	0		1			
	XP_015777540.1	PREDICTED: integrin alpha-PS1-like [*Acropora digitifera*]		○	−3300.3	−3312.8	−3313.28	25.1	<0.0001*	3 codon sites	−3312.8	−3312.8	0		1			
	XP_015750743.1	PREDICTED: integrin alpha-8-like [*Acropora digitifera*]		○	−1402.2	−1403.2	−1403.3	2	0.16		−1401.8	−1403.2	2.98		0.08			
	XP_015763542.1	PREDICTED: integrin-linked protein kinase-like [*Acropora digitifera*]	○	○	−740.1	−741.1	−742.89	1.96	0.16		−740.6	−740.6	0		1			
	XP_015757723.1	PREDICTED: calcium and integrin-binding protein 1-like [*Acropora digitifera*]		○	−402.1	−402.1	−402.09	0	1		−402.1	−402.1	0		1			
	XP_015777529.1	PREDICTED: integrin alpha-9-like, partial [*Acropora digitifera*]		○	−2635.8	−2670.5	−2670.58	69.4	<0.0001*	22 codon sites	−2698.2	−2712.1	27.89		<0.0001*	16S, 82M, 160F		positive slections in forregroud was not supported
	XP_015772241.1	PREDICTED: integrin beta-PS-like [*Acropora digitifera*]		○	−2334.3	−2335.9	−2336.26	3.19	0.073654301*	2 codon sites	−2335.5	−2335.8	0.6		0.44			
ADAMs																		
	XP_015757289.1	PREDICTED: A disintegrin and metalloproteinase with thrombospondin motifs 6-like [*Acropora digitifera*]		○	−1719.3	−1721.5	−1721.76	4.25	0.039*		−1720	−1721.45	2.89		0.089			
	XP_015769952.1	PREDICTED: disintegrin and metalloproteinase domain-containing protein 12-like isoform X1 [*Acropora digitifera*]		○	−6259.9	−6277.2	−6277.32	34.5	<0.0001*	5 codon sites	−6277.2	−6277.2	0		1			
	XP_015758816.1	PREDICTED: A disintegrin and metalloproteinase with thrombospondin motifs 18-like [*Acropora digitifera*]		○	−2181.6	−2183.4	−2181.61	3.74	0.053		−2181.3	−2183.1	3.56		0.059			
	XP_015769077.1	PREDICTED: A disintegrin and metalloproteinase with thrombospondin motifs 6-like [*Acropora digitifera*]		○	−918.5	−920.8	−920.8	4.51	0.034*	1 codon site	−920.5	−920.5	0		1			
	XP_015778638.1	PREDICTED: disintegrin and metalloproteinase domain-containing protein 10-like [*Acropora digitifera*]	○	○	−2227.3	−2240.5	−2240.82	26.4	<0.0001*	2 codon sites	−2240.4	−2240.4	0		1			
	XP_015778639.1	PREDICTED: disintegrin and metalloproteinase domain-containing protein 10-like [*Acropora digitifera*]	▲	○	−3608.5	−3663.1	−3663.22	109.2	<0.0001*	15 cidin sites	−3270.3	−3277.9	15.21		<0.0001*	56R, 386D		Not supported
	XP_015765941.1	PREDICTED: disintegrin and metalloproteinase domain-containing protein 10-like, partial [*Acropora digitifea*]		○	−1960.9	−1964.7	−1964.71	7.61	0.0058*	1 codon site	−1964.7	−1964.7	0		1			
	XP_015780892.1	PREDICTED: ADAM 17-like protease [*Acropora digitifera*]		○	−2941.9	−2987.7	−2989.94	91.6	<0.0001	35 codon sites	−3329.8	−3333.4	7.4		0.0065*	103S		Not supported
Tetraspanins																		
	XP_015766542.1	PREDICTED: tetraspanin-3-like [*Acropora digitifera*]		○	−817.2	−831.9	−832.27	29.6	<0.0001*	13 codon sites	−1496.7	−1497.9	2.33		0.126			
	XP_015766319.1	PREDICTED: tetraspanin-4-like [*Acropora digitifera*]	○	○	−743.9	−751.6	−751.65	15.5	<0.0001*	4 codon sites	−996.9	−1002.5	11.08		<0.0001*	191E		Supported in A.muricata
	XP_015756085.1	PREDICTED: tetraspanin-33-like [*Acropora digitifera*]	○	○	−116.09	−117.29	−117.3	2.41	0.12		−117.3	−117.3	0					
	XP_015764299.1	PREDICTED: tetraspanin-7-like [*Acropora digitifera*]	○	○	−1320.8	−1327.1	−1327.31	12.51	<0.0001*	2 codon site	−1327.1	−1327.1	0					
	XP_015759357.1	PREDICTED: CD63 antigen-like [*Acropora digitifera*]	○	○	−1349.5	−1365.9	−1365.92	32.8	<0.0001*	8 coden sites	−1365.7	−1365.7	0					
	not registrated	CD9		●	−1252.4	−1253.5	−1253.87	2.21	0.14		−1253.5	−1253.5	0					

▲: not detected in western blotting but identiferd in mass analyses.

●: not identified in mass analyses but detected in western blotting.

Tetraspanins were found in eggs (tetraspanin 3, 4, 7, and 33) and sperm (tetraspanin 4, 7, and CD63). CD9 was not detected in either eggs or sperm; CD9 of *A. digitifera* is not registered in GenBank (https://www.ncbi.nlm.nih.gov/gene/); CD9 could not be identified due to lack of the data. However, CD9 of *Acropora* was obtained from Orthoscope, and cDNA of CD9 in *A. digitifera* was isolated*.* We prepared an antibody from the cDNA of *A. digitifera*, and the antibody reaction was observed corresponding to its mass. Therefore, CD9 is plausibly present in *Acropora* eggs.

### 3.2 Molecular evolution of integrins, ADAMs, and tetraspanins

Codon site analysis suggested several genes were positively selected among congeneric *Acropora* (Table 1). Most species whose genome information is available to show synchronous spawning and gamete compatibility are also identified, except for several species (Table 2). For example, *A. digitifera* does not show crossing among species in the database for Orthoscope. Branch site analyses were conducted to specify the correlation between the substitution rates of the several limited amino acid sites and species-specific fertilization. Of the integrins, ADAMs, and tetraspanins, four genes (*ADAM10*, *ADAM17*, *integrin α9*, and *tetraspanin 4*) had positively selected sites during the acquisition of fertilization specificity (Table 1). In contrast, aBSREL analyses support branch analyses only in *A. muricata* in the tetraspanin 4 (Table 1). *CD9* was subjected to strong purifying selection.

**TABLE 2:** Spawning synchronisms and gamete compatibility in the *Acropora* of the database of Orthoscope.

Species	Spawning	Spawning time	Gamete compatibility	
*Acropora digitifera*	June	21:40–22:30		*Acropora papillare* (not in the database) is compatible.
*Acropora tenuis*	June	19:20–19:40	^a^	*Acropora donei* (not in the database) is also compatible.
*Acropora awi*	unknown	unknown		
*Acropora echinata*	unknown	unknown		
*Acropora nasuta*	June	22:10–22:30		
*Acropora gemmifera*	June	22:10–22:40	^b^	
*Acropora intermedia*	June	22:20–22:40	^b^ ^c^	
*Acropora florida*	June	21:40–22:10	^b^ ^c^	
*Acropora muricata*	June	22:17–22:23		
*Acropora yongei*	June	19:30 (from Fukami et al., 2003)	^a^	
*Acropora hyacinthus*	June	22:20–22:40		
*Acropora cytherea*	June	22:20–22:40		
*Acropora mircophthalma*	unknown	unknown		
*Acropora acuminata*	June	22:10–22:30		
*Acropora selago*	unknown	unknown		

^a^ Compatible each other.

^b^ Compatible each other.

^c^ Compatible each other.

**FIGURE 1:**
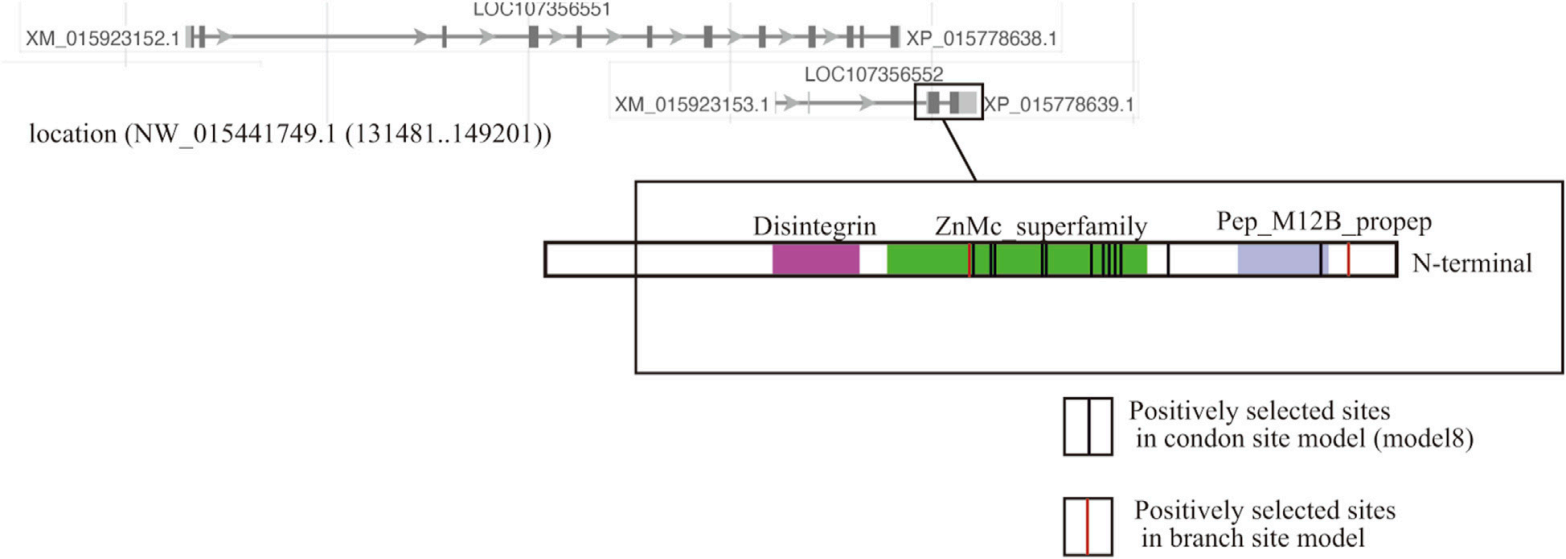
Localization of two copies of ADAM10 on the genome of the coral Acropora digitifera. Of the two ADAM10 sequences (XP_015778638.1, XP_015778639.1), the substitution rates of the several limited amino acid sites in one copy (XP_015778639.1) was accelerated. The red lines show codon sites in the Bayes empirical Bayes (BEB) analyses in the branch site model, and the black lines indicate the codon site model (Table 1).

Two *ADAM10* sequences were detected (XP_015778638.1, XP_015778639.1), but only one *ADAM10* (XP_015778639.1) supported the branch-site model according to the species-specific fertilization mechanism. The loci of these two genes overlapped, but positive selection sites of *ADAM10* (XP_015778639.1) were localized on a distinctive region of the genome (Figure 1).

### 3.3 Involvement of the branch-site supported integrins, ADAMs, and tetraspanins in fertilization

**FIGURE 2:**
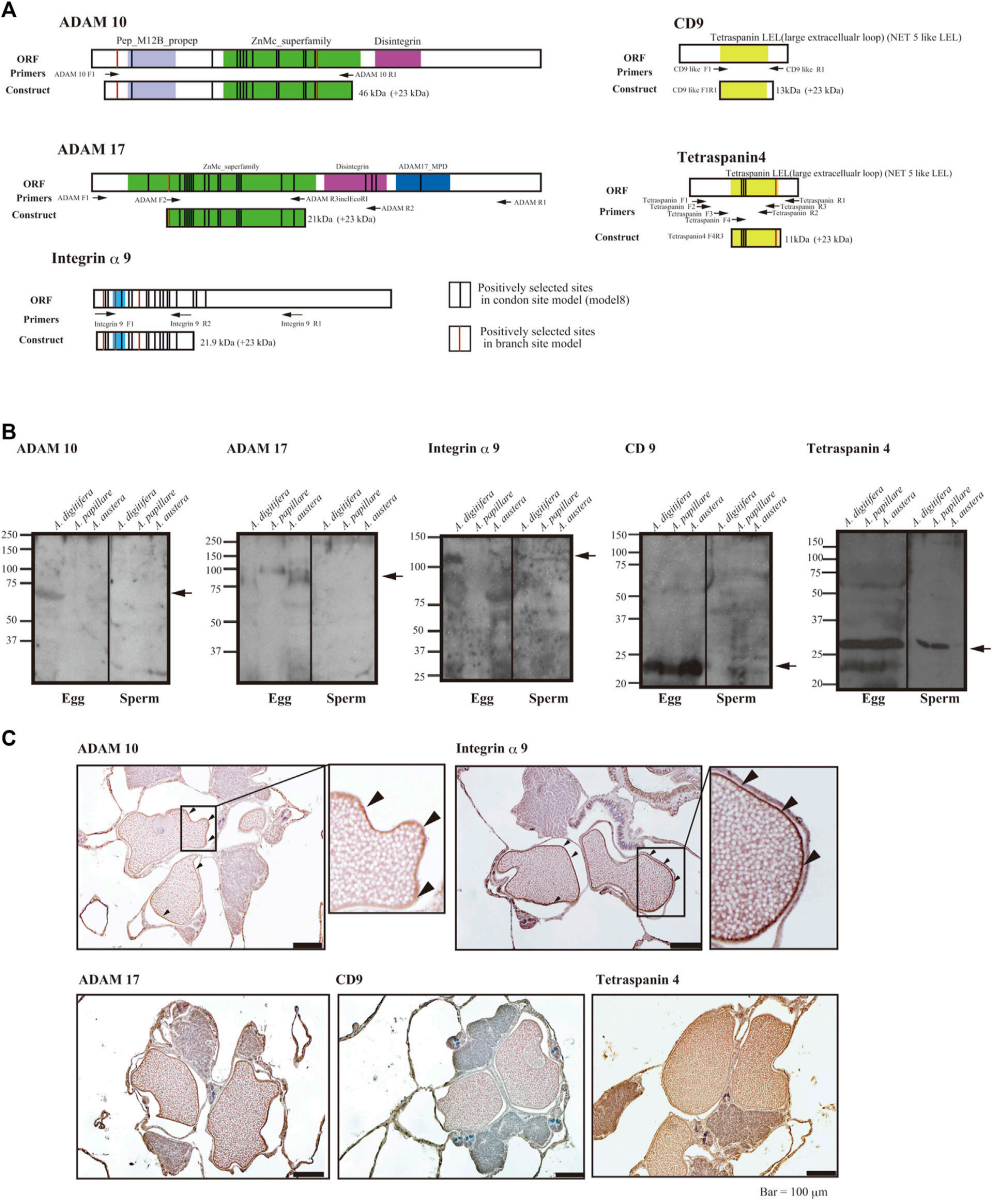
**(A)** Functional domains and positively selected sites of integrin α9, tetraspanins, ADAMs, and region(s) of antigen for antibody generation, **(B)** immunoblot analyses, and **(C)** immunohistology with the antibodies against sperm and eggs of the coral *Acropora.* **(A)** Functional domains of open reading frames, location of primers, and positively selected sites are indicated (red indicates the branch site model and black, the codon site model). Upper panels are hydropathy plots of deduced amino acid sequences. **(B)** Immunoblot analyses with eggs or sperm of *A. digitifera*, *A. papillare*, and *A. austera*. For tetraspanin 4 and CD9, 10% acrylamide gel was used to separate the proteins, and 7.5% gel was used for ADAMs and integrin α9. **(C)** Localization of ADAM10, ADAM17, Integrin α9, CD9, and Tetraspaning in about 1 week before the spawning of the coral *A. digitifera*. Arrowheads indicate the surface of oocytes. Bars = 100 μm.

We developed antibodies against the proteins (integrin α9, ADAM10, ADAM17, and tetraspanin 4), for which the substitution rates of the several amino acid sites had been accelerated according to species-specific fertilization traits. We prepared antibodies against the antigen of each protein containing the positively selected sites (Figure 2A). Immunoblotting using each antibody suggested that ADAMs, integrins α9, and CD9 were present only in the eggs, but tetraspanin4 was localized in both sperm and eggs (Figure 2B). All of the bands corresponded the expected molecular mass from CDs sequences (ADAM10; 68 kDa, ADAM17; 94 kDa, Integrin α9; 110 kDa, tetraspanin 4; 26 kDa, CD9; 28 kDa). In anti-ADAM10 and integrin α9 antibodies, antibodies reacted only with proteins in *A. digitifera* eggs (Figure 2B). Immunohistochemistry also showed that ADAM10 was localized on the surface of the oocytes (Figure 2C). ADAM17 localized both oocytes and strong signal was found on the surface of the oocytes like ADAM10 and integrin α9. CD9 were mainly found in the oocytes but tetraspanin 4 were stained many tissues including oocytes.

**FIGURE 3:**
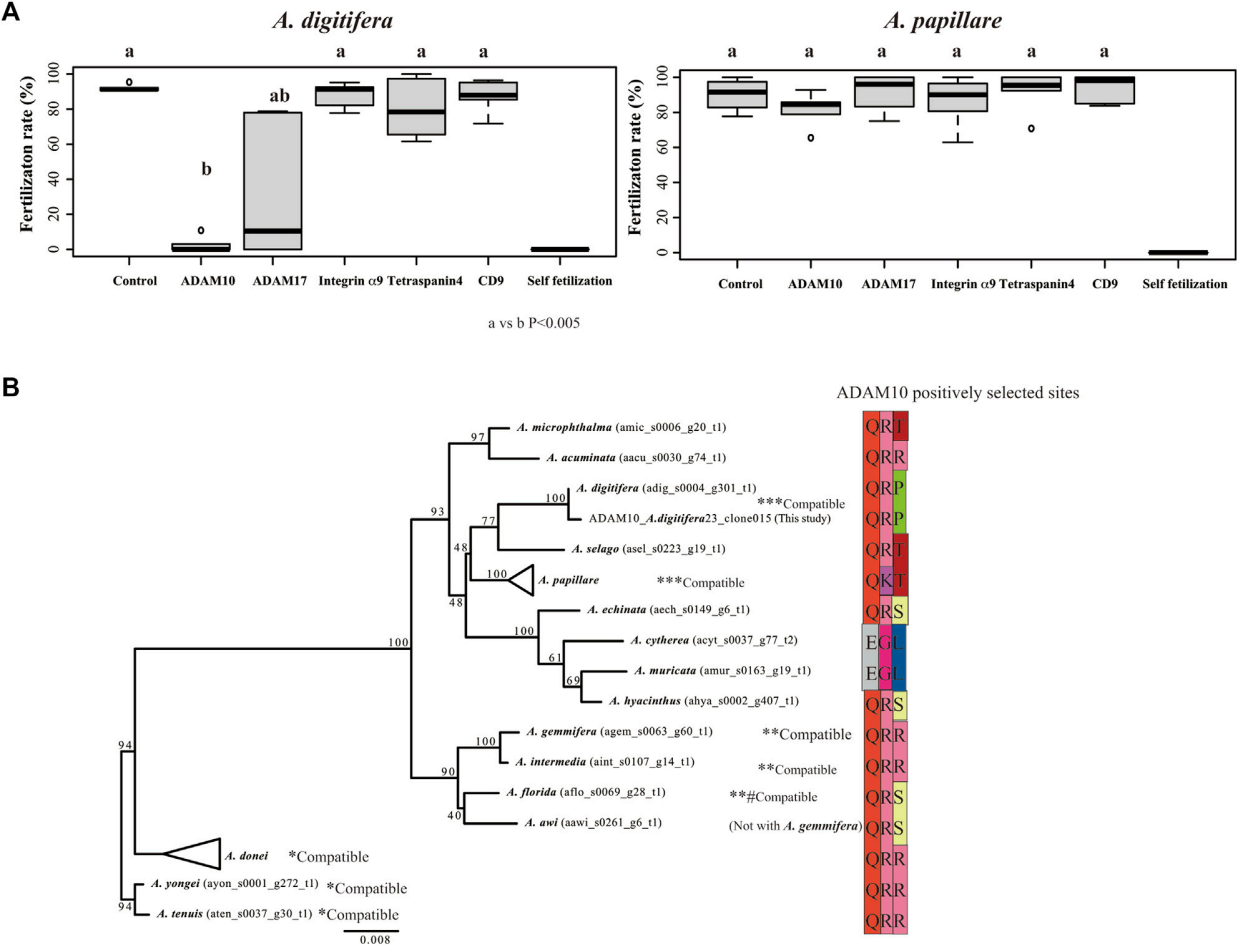
Fertilization ratio in the presence of antibodies against tetraspanins, integrin α9, and ADAMs. Fertilization in the presence of 10 μL of each antibody (1 mg/mL IgG) in 1 mL seawater (finally 10 μg/mL) was observed in gametes from *Acropora digitifera* and *A. papillare*. **(A)** Fertilization was conducted at 106 sperm/mL conditions, and the mixture of the gametes was finished within 2 h of spawning. Six crosses using gametes from three colonies of each species (*N* = 6; three colonies from both *A. digitifera* and *A. papillare*). Self-fertilization was used as a negative control. **(A)** or **(B)** indicates a significant difference (Wilcoxon rank sum test) **(B)** Maximum likelihood (ML) phylogenetic tree of *ADAM10* and sequences of positively selected sites (branch site analyses) among species. Gamete-compatible species are indicated with *, **, or ***.

Of the candidates that mediated species-specific fertilization, only antibodies against ADAM10 strongly inhibited fertilization in *A. digitifera* (Figure 3A; vs. control, *p* < 0.001). ADAM17 slightly inhibited fertilization in *A. digitifera* (Figure 3A; vs. control, *p* < 0.05). Other antibodies against integrin α9, tetraspanin4, and CD9 did not inhibit fertilization in any tested species, including *A. papillare* (Figure 3A; *p* > 0.05). The positively selected sites of ADAM10 in *Acropora*, including *A. papillare*, were different (Figure 3B). As a control, we did intercross experiments between *A. digitifera* and *A. papillare*, showing intercrossing but not fully compatible (Supplementary Figure S2A). In the *A. austera*, we could succeed with experiments only with one combination. We thus could not conclude anything from the results (Supplementary Figure S2B). However, the fertilization was not suppressed with antibodies except CD9. The gametes of all colonies did not show self-fertilization as a negative control (Figure 3A).

## 4 Discussion

In this study, we examined the roles of integrins, ADAMs, and tetraspanins in *Acropora* fertilization. As shown in mammals, the roles of ADAMs and integrins are controversial (e.g., Evans, 2001; He et al., 2003). Integrin β1 has been reported to be involved in *Acropora* fertilization (Iguchi et al., 2007). The other integrins, ADAMs, and tetraspanins were found in the gametes of *A*. *digitifera*. In the presence of antibodies against ADAM10, fertilization in *A. digitifera* was inhibited; ADAM10 may therefore be associated with fertilization in *A. digitifera*.

Although ADAM10 has not been reported to be involved in fertilization, our results suggest that ADAM10 is partly associated with gamete species recognition in *Acropora*. Fertilization-related ADAMs (ADAM2, ADAM9, ADAM12, ADAM15, and ADAM23) (Evans, 2001) interact with integrins (alpha4beta3, alpha9beta1) (Vjugina et al., 2009; Desiderio et al., 2010). The differences in the architecture of eggs between coral and mice suggest that the functions of ADAMs differ among species. For example, ADAMs and integrins are suggested to contribute to gamete interaction only when the ZP is present in the eggs (Evans et al., 1997). In *Acropora*, there is no ZP, and ADAM10 was found only in the eggs; the interacting integrins were not found in the sperm. Antibodies against integrin β1 slightly inhibit fertilization (Iguchi et al., 2007), and thus, the involvement of integrin β1 should be carefully considered. Overall, ADAM10 may interact with proteins other than integrins. Further studies are required to identify the protein(s) that interact with ADAM10.

The positively selected codon site in ADAM10 differs among species. Although the ADAM10 antibody specifically inhibited *A. digitifera* fertilization, only the two positively selected codon sites differed between *A. papillare* and *A. digitifera*. In addition, only two positively selected codon sites may be insufficient to determine their fertilization specificity for more than 20 synchronous species (Baird et al., 2021) or 110 extant species. Therefore, whether ADAM10 governs fertilization specificity among at least 20 synchronous spawning species is questionable. In addition, the feasibility of intercrossing between *A. digitifera* and *A. papillare* provokes the hypothesis that other gamete-composing proteins also play a role in species recognition.

Positive selections for species recognition might have arisen in many gamete protein genes in the Acropora spp. (Table 1; Morita et al., unpublished data). Specific codon sites are positively selected to generate polymorphic regions for recognition. Gamete compatibility is often congruent with rates of molecular evolution (Zigler et al., 2005). In addition, the risk of hybridization could influence the rates of molecular evolution. For example, the mixture of gametes from congeneric species could be associated with the risk of hybridization. The rates of codon evolution become slower when gamete interactions among heterospecifics are rare (Geyer et al., 2020).

The fertilization specificity and spawning synchronicity of several species (e.g., *A. awi* and *A. echinata*) used in this study are unknown. Thus, these species were not set as “foreground” for branch site analyses. For expedience, these species with unidentified fertilization specificity, such as *A. awi* and *A. echinate*, were set as background sequences. Due to the inclusion of these ambiguous species, the branch site analyses possibly underestimate the positively selected sites, and the robustness of the analyses needs to be considered carefully.

Coral is a basal animal, and its fertilization mechanisms are likely different from those in mammals, except for the plasma membrane fusion between sperm and egg. During the membrane fusion of sperm and eggs, CD9 functions as a strong determinant of fertilization in the mouse pathway (Miyado et al., 2008). Therefore, antibodies against CD9 were expected to suppress fertilization in all examined species. In contrast to our prediction, the CD9 antibody did not suppress fertilization. We developed an antibody against *A. digitifera* CD9 using its long extracellular loop (LEL) region, which is suggested to be involved in sperm–egg fusion (Umeda et al., 2020). It is also possible that antibodies against *A. digitifera* CD9 do not react with the functional site of CD9. In addition, small vesicles containing CD9 are released during membrane fusion between sperm and eggs in mice (Miyado et al., 2008; Barraud-Lange et al., 2012), but coral eggs are filled with wax esters (Harii et al., 2007), which are too rigid to form vesicles.

Another possibility is that CD9 does not underlie membrane fusion between sperm and eggs of the coral *Acropora*. The partner of CD9, EWI-2 protein with an Ig domain (Stipp et al., 2001), was not found in the eggs. Although EWI-2 protein has not been identified by MS analysis due to non-registration of EWI-2 cDNA, treatment with an anti-CD9 antibody against its LEL region was presumably insufficient to suppress the membrane fusion process if CD9 worked together with the EWI-2 complex. Our preliminary data found that proteins with IgG domains may be related to fertilization, but whether a protein–IgG complex interacts with CD9 and facilitates membrane fusion is unclear. To identify the differences between mammals and coral, the detailed pathway from sperm adhesion to membrane fusion should be investigated in *Acropora*.

The functions of the other tetraspanins are predicted to be different because differences in egg architecture are associated with differences in gamete adhesion pathways (e.g., Frolikova et al., 2019). Tetraspanins related to plasma membrane fusion (CD81 and CD151) were not found in the coral sperm. However, CD63, which functions as a primary adhesive of the cumulus cell layer in mammalian eggs, was found. In this study, we did not examine the role of CD63 because the positive selection of *CD63* was not supported (Table 1). CD63 in mouse sperm is implicated in sperm–egg interactions via integrins (Frolikova et al., 2019). Functional investigation of other tetraspanins (including CD63) is needed to identify the evolution and differentiation of fertilization mechanisms through speciation.

Among the identified gamete proteins, the rapid evolution of integrin α9, ADAMs, and tetraspanin 4 is supported by molecular evolutionary analysis. Functional modifications might occur in these proteins, and their functions are potentially not limited to fertilization. In this study, although the immunostaining implies these proteins may be present in the many tissues in the corals, we did not investigate the localization of these proteins in other tissues. Therefore, their localization and roles in different tissues are still unclear. Their functions could be diverse and potentially useful in many tissues. Indeed, the ADAM10 and 17 localize many tissues and function in many aspects, such as the embryonic development process (Harrison et al., 2021). Although the function of integrins and ADAMs are questionable, integrins α9 forms complex with could interact with CD9 in the mouse (Zhu and Evans, 2002). *Acropora* has experienced climate change and is surviving. The rapid evolution of the proteins is presumably associated with the tolerance/resilience of the corals. However, further study is required.

In conclusion, we examined the roles of integrins, ADAMs, and tetraspanins in fertilization. As a result of this study, ADAM10 plausibly mediates species recognition. The ADAM10 antibody reacted only with *A. digitifera*, and thus, it still needs to be confirmed that ADAM10 governs species-specific fertilization in the other *Acropora* spp. In addition, positively selected sites in ADAM10 that arose during the acquisition of strict species recognition are limited. Overall, we predict more proteins are involved in species recognition, which is deeply associated with species boundaries in the coral *Acropora*.

## Data Availability

The datasets presented in this study can be found in online repositories. The names of the repository/repositories and accession number(s) can be found below: https://zenodo.org/deposit/6483451, 10.5281/zenodo.6483451.
