# Supplementary material for: Positive selection on ADAM10 builds species recognition in the synchronous spawning coral Acropora
Source: Front Cell Dev Biol. 2023 Apr 20;11:1171495. doi: 10.3389/fcell.2023.1171495 (PMC10157049; doi:10.3389/fcell.2023.1171495)
Supplement: Supplementary file 1 [file DataSheet2.PDF]

Codon site morel

Codon evolved at same rate (ω>1)

Model8 (ω>1) vs model8a (ω = 1)

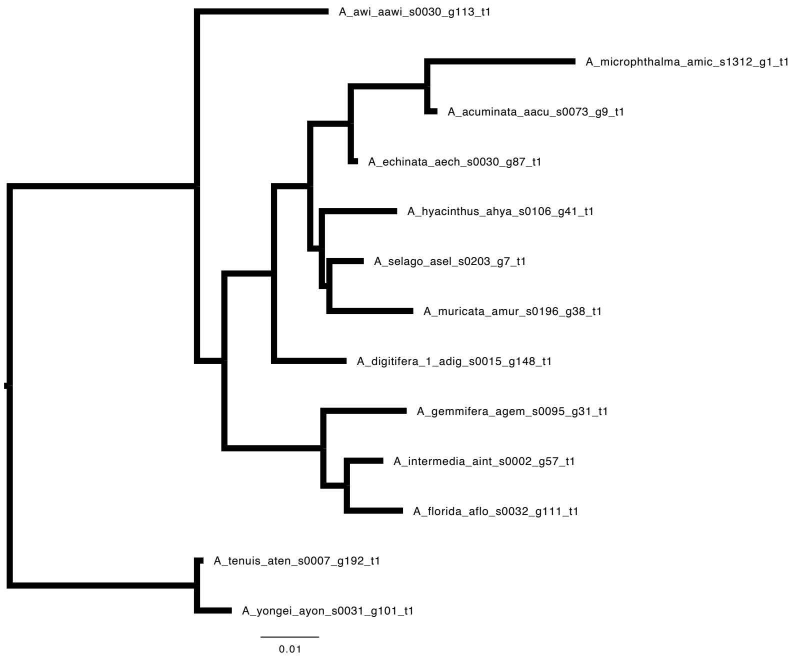

Branch site model

Specific branch (red) shows accelerated evolution.

Model2a (ω > 1) vs null (ω = 1)

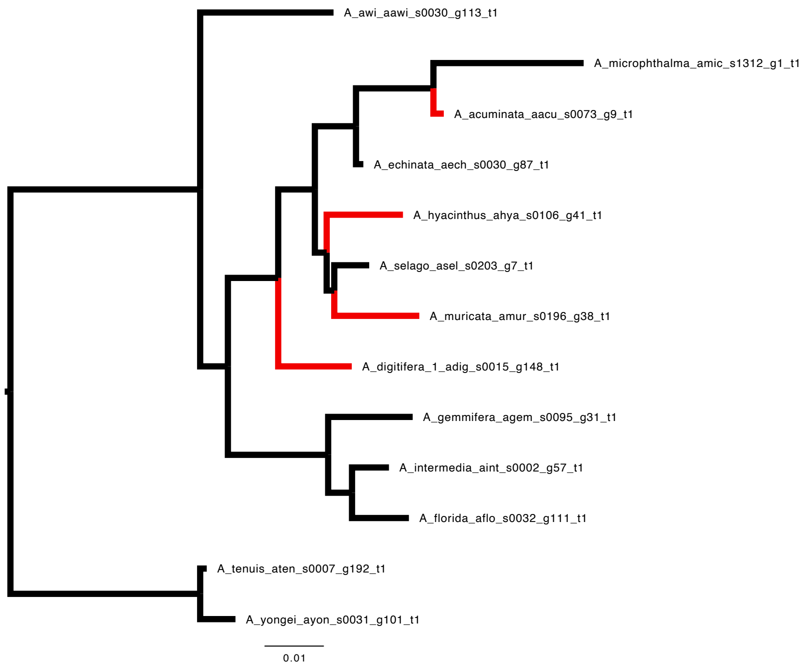

Supplementary figure 1 Codon site and branch-site models. In the codon site model, evolutionary rates are presumed to be constant. In contrast, non-crossing species are categorized as foreground branches (red).
