# Supplementary figures and images for: Positive selection on ADAM10 builds species recognition in the synchronous spawning coral Acropora
Source: Front Cell Dev Biol. 2023 Apr 20;11:1171495. doi: 10.3389/fcell.2023.1171495 (PMC10157049; doi:10.3389/fcell.2023.1171495)

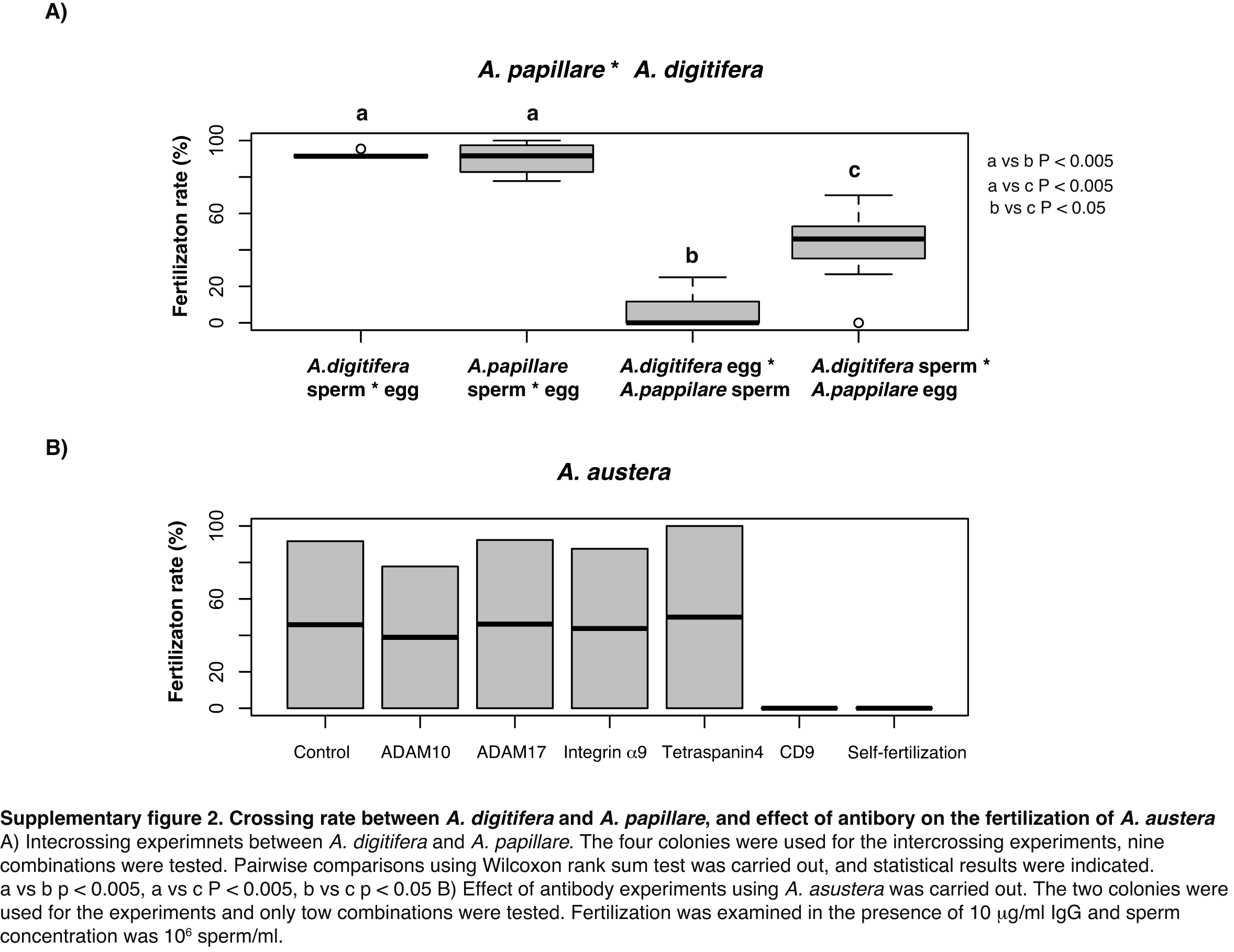

Supplement: Supplementary file 2 [file Image1.TIF]
