# Supplementary material for: Positive selection on ADAM10 builds species recognition in the synchronous spawning coral Acropora
Source: Front Cell Dev Biol. 2023 Apr 20;11:1171495. doi: 10.3389/fcell.2023.1171495 (PMC10157049; doi:10.3389/fcell.2023.1171495)
Supplement: Supplementary file 3 [file DataSheet1.PDF]

Supplementary Table 1. Primers for TA cloning and making constrcuts

Targets gene	Primer name	Sequence
**CD9**	Acropora_CD9like F1	TACATTTATAGAGAAGACATTGAGAAGAA
	Acropora_CD9like R1	ACTTATCACATGCAAGTTATTAAGAAGG
	AAA_XhoI_Acropora_CD9like F1	AAActcgagTACATTTATAGAGAAGACA
	AAA_EcoRI_Acropora_CD9like R1	AAAgaattcACTTATCACATGCAAGT
**Tetraspanin 4**	Tetraspanin_4 F1	gcagtggaaggagca(AorG)(CorT)aag
	Tetraspanin_4 F2	AGATGTGTAAAAATTCTCATGTTTGTT
	Tetraspanin_4 F3	AAAGAAAACAGATGTCTTCTTGGA
	Tetraspanin_4 F4	acatcattgggctatgtgtatcg
	Tetraspanin_4 R1	GGCATATGTGTTTTGGTCTTTG
	Tetraspanin_4 R2	CTTGTTCC(CorA)(TorC)CTGAAATGTTTC
	Tetraspanin_4 R3	ggtcttctcaaaacatccttcagt
	AAA_XhoI_Tetraspanin_4_F1	AAActcgagGcagtggaag
	AAA_EcoRI_Tetraspanin_4_R1	AAAgaattcGGCATATGTGTTTTG
	AAA_XhoI_Tetraspanin_4 F2	AAActcgagAGATGTGTAAAAATTC
	AAA_XhoI_Tetraspanin_4 F3	AAActcgagAAAGAAAACAGATGTC
	AAA_XhoI_Tetraspanin_4 F4	AAActcgagACATCATTGGGC
	AAA_EcoRI_Tetraspanin_4 R2	AAAgaattcCTTGTTCC(CorA)(TorC)CTG
	AAA_EcoRI_Tetraspanin_4 R3	AAAgaattcGGTCTTCTCAAAACATC
**Integrin 9α**	integrin_alpha_9_F1	GAAGGCGAAGATGGAAGC
	integrin_alpha_9_R1	ATCAGCTTCATACTTGACGGC
	integrin_alpha_9_R2	ATCAAAAGGGAGAAGAGAGGTGA
	AAA_XhoI_integrin_alpha_9_F1	AAActcgagGAAGGCGAAG
	AAA_EcoRI_integrin_alpha_9_R1	AAAgaattcATCAGCTTCATACTTG
	AAA_EcoRI_integrin_alpha_9_R2	AAAgaattcATCAAAAGGGAGAAGAGA
**ADAM10**	ADAM10 F1	GCATTGTCATACCGACGAC
	ADAM10 R1	GTTTCTCTTATCTCCACTCGT(GorA)G
	AAA_XhoI_ADAM_10_F1	AAActcgagGCATTGTCATAC
	AAA_EcoRI_ADAM_10_R1	AAAgaattcGTTTCTCTTATCTCCA
**ADAM17**	ADAM17-like F1	GCATATCGACATTC(AorT)GATGTGAA
	ADAM17-like F2	AGAACGGAGTGGTCAAATGAATA
	ADAM17-like R1	CCTCTT(AorG)TCTCTCCATTC(GorC)GC
	ADAM17-like R2	CTCTTTCTTGCAAGATG(CorA)G
	ADAM17-like R3inclEcoRI	CAAgaattcTCCGCTTGCT
	AAA-XhoI-ADAM17-like F1	AAActcgagGCATATCGACATTC
	AAA-XhoI-ADAM17-like F2	AAActcgagAGAACGGAGTG
